# Constant Resting Frequency and Auditory Midbrain Neuronal Frequency Analysis of *Hipposideros pratti* in Background White Noise

**DOI:** 10.3389/fnbeh.2021.657155

**Published:** 2021-05-25

**Authors:** Guimin Zhang, Zhongdan Cui, Jing Wu, Baoling Jin, Dandan Zhou, Long Liu, Jia Tang, Qicai Chen, Ziying Fu

**Affiliations:** ^1^Hubei Key Laboratory of Genetic Regulation and Integrative Biology, School of Life Sciences, Central China Normal University, Wuhan, China; ^2^College of Science, National University of Defense Technology, Changsha, China

**Keywords:** echolocating bat, background noise, resting frequency, frequency tuning, auditory midbrain, Lombard effect

## Abstract

Acoustic communication signals are inevitably challenged by ambient noise. In response to noise, many animals adjust their calls to maintain signal detectability. However, the mechanisms by which the auditory system adapts to the adjusted pulses are unclear. Our previous study revealed that the echolocating bat, *Hipposideros pratti*, increased its pulse intensity in the presence of background white noise. *In vivo* single-neuron recording demonstrated that the auditory midbrain neurons tuned to the second harmonic (H2 neurons) increased their minimal threshold (MT) to a similar degree as the increment of pulse intensity in the presence of the background noise. Furthermore, the H2 neurons exhibited consistent spike rates at their best amplitudes and sharper intensity tuning with background white noise compared with silent conditions. The previous data indicated that sound intensity analysis by auditory midbrain neurons was adapted to the increased pulse intensity in the same noise condition. This study further examined the echolocation pulse frequency and frequency analysis of auditory midbrain neurons with noise conditions. The data revealed that *H. pratti* did not shift the resting frequency in the presence of background noise. The auditory midbrain neuronal frequency analysis highly linked to processing the resting frequency with the presence of noise by presenting the constant best frequency (BF), frequency sensitivity, and frequency selectivity. Thus, our results suggested that auditory midbrain neuronal responses in background white noise are adapted to process echolocation pulses in the noise conditions.

## Introduction

Humans and many animals rely on acoustic information for survival and breeding. However, acoustic signal transformations are inevitably influenced by biotic and abiotic noise. In response to noise, humans and animals recruit numerous strategies to maintain their ability to detect acoustic signals (Brumm and Slabbekoorn, 2005; Corcoran and Moss, 2017). Among these strategies, some are long-term evolutionary responses. For example, urban great tits (*Parus major*) tend to sing at a higher pitch in high-level low-frequency locations compared with birds in quieter areas (Slabbekoorn and Peet, 2003). Many animals also have evolved several short-term adaptive modifications. For instance, the famous Lombard effect, which refers to an involuntary rise in call amplitude to ambient noise (Brumm and Zollinger, 2011). The Lombard effect was first described in humans and was also observed in fish, amphibians, birds, and mammals, including echolocating bats (Luo et al., 2018).

Accompanying the Lombard effect, many animals also shift the spectral-temporal composition of their vocalization to avoid noise interference, known as noise-induced vocal modification (Hotchkin and Parks, 2013). Although most published papers have reported that echolocating bats exhibited the Lombard effect in response to noise, the changes in the spectral composition were not as widespread as amplitude. Previous results from bats producing broadband frequency-modulated (FM) echolocation pulses (FM bats) indicated that they tended to exhibit a linkage between the Lombard effect and shifts in spectral-temporal composition (Schmidt and Joermann, 1986; Tressler and Smotherman, 2009; Tressler et al., 2011). As such, the shift in spectral-temporal composition was interpreted as a “by-product” of the Lombard effect (Tressler and Smotherman, 2009). However, studies on the horseshoe bat (*Rhinolophus ferrumequinum*), a constant frequency-FM (CF-FM) bat, showed that shifts in pulse amplitude and spectral composition were controlled independently (Hage et al., 2013, 2014). *R. ferrumequinum* is a long CF-FM bat, and its echolocation pulses are characterized by long CF components, which are typically 30–70 ms (Schuller and Pollak, 1979). A recent study on short CF-FM bats from the Hipposideridae family reported that their rest frequencies (RFs) were relatively stable across all noise levels tested (Lu et al., 2020). Since Hage et al. (2013) and Lu et al. (2020) worked on different family of bat species (Rhinolophidae vs. Hipposideridae) and broadcasted noise of distinct frequency ranges, the driving factors for the reported differences in noise-induced modifications of the RF between these two studies were unclear.

The auditory system is adapted to deal with behavior related sounds. For example, the male bush cricket exhibited a significantly expanded auditory fovea tuned to the dominant frequency of the female reply to facilitate their ability to find a mate (Scherberich et al., 2017). The auditory sensitivity of FM and CF-FM bats also closely corresponds to the frequency range of their orientation pulses (Neuweiler, 2003). For example, the auditory system of CF-FM bats is specialized to process the RF of their echolocation pulses (Schnitzler and Denzinger, 2011). Although a number of noise-induced pulse adjustments in the echolocation bat have been uncovered (Corcoran and Moss, 2017), whether and how the auditory system is modified to adapt to processing pulses in noise conditions is largely unknown.

In a recent study on a short CF-FM bat, *Hipposideros pratti*, we reported that their pulse intensity increased in response to 70-dB SPL background white noise, while *in vivo* single-neuron recording revealed that the auditory midbrain neurons tuned to the second harmonic (H2 neurons) increased their minimal threshold (MT) to a similar degree as the increment of pulse intensity in the presence of the background noise. Furthermore, the H2 neurons exhibited consistent spike rates at their best amplitudes and sharper intensity tuning with background white noise compared with silent conditions. The previous data indicated that sound intensity analysis by auditory midbrain neurons was adapted to the increased pulse intensity in the same noise condition (Cui et al., 2021). The present study further examined the effect of background white noise on the spectral composition of their echolocation pulses and midbrain neural frequency sensitivity. We report here that the noise has no significant influence on the RFs of the echolocation pulses. The auditory midbrain neuronal frequency analysis is highly linked to processing the resting frequency during noise with constant best frequency (BF), frequency sensitivity, and frequency selectivity. Thus, this study and previous results (Cui et al., 2021) indicated that auditory midbrain neuronal responses during background white noise were adapted to processing echolocation pulses in the noise.

## Materials and Methods

### Ethics Approval

All experiments were conducted with the approval of the Institutional Animal Care and Use Committee of Central China Normal University, Wuhan, Hubei, PRC (Permit Number: ccnu2017640-0066). All surgeries and recordings were performed under sodium pentobarbital anesthesia, and all efforts were made to minimize suffering.

### Animal Preparation

Six *H. pratti* (four males and two females; 40.8–69.8 g, body weight) were captured in a cave (N: 29° 26′ 0.32″; E: 114° 01′ 20.49″) near Xianning City of Hubei province, China, and used in this study. The bats were captured using a ground-level mist net. A mist net (2.0 m × 3.0 m) was opened for 6 h from dusk until midnight (the mist net was inspected every 10 min). The six bats were housed socially in an animal room (dimensions: 3.0 m × 3.0 m × 3.0 m) and exposed to local photoperiod, as well as constant temperature (28–30°C) and humidity (>60% relative humidity). The bats had free access to water and food (mealworm) *ad libitum*. The bats were examined daily for any sign of weakness or illness, including inappetence or slow responses. Bats in poor physiological condition were excluded from specific experiment on that day and put back in the animal room. The Forestry Department of Hubei Province provided permission to conduct the study on this site. No additional permissions were required for our research because *H. pratti* is not considered an endangered or protected species.

### Recordings of Echolocation Pulses

The recordings of the echolocation pulses were performed several days after each bat was acclimated to the laboratory conditions. All recordings were conducted at approximately 7 p.m. during June, 2018. First, each bat was trained to hang stably on the ceiling of the experimental anechoic cage. During each recording, the recording microphone was placed 1 m below the bat in the bat’s frontal azimuth space. The microphone’s membrane was pointed toward the bat’s nose as best as possible. The echolocation pulses were recorded from each bat in isolation using an ultrasound detector (Petterson D1000X; Pettersson Elektronik AB, Uppsala, Sweden) at a sampling rate of 384 kHz. Each bat was recorded one to three times for 20 s, with a 5-s silence control, 10 s with 70-dB SPL white noise, and 5-s silence after the noise. The gain control was adjusted to avoid saturation of the recording system.

The echolocation pulse recordings were also performed before each electrophysiological recording to quantify the frequency spectrum of the H2 for each bat’s echolocation pulse. During the recordings, each bat was hanging on the ceiling of the experimental anechoic room and the recording microphone was placed 1 m below the bat in the bat’s frontal azimuth space. The echolocation pulses were recorded from each sedentary bat in real time using a handheld ultrasound detector (Petterson D1000X; Pettersson Elektronik AB, Uppsala, Sweden) before being entered into a notebook computer. Each bat was recorded one to three times, and each time lasted 3–5 s.

### Pulse Analysis

The echolocation pulses were analyzed using the BatSound pro 3.31b (Pettersson Elektronik AB, Uppsala, Sweden), with a fast Fourier transformation (FFT) size of 8,192 points and a Hanning window with a cursor and visual determination on a screen. The sound frequencies were collected from the power spectrum.

### Animal Surgery

The surgical procedures were similar to those described in our previous studies (Fu et al., 2010, 2019). Briefly, 1 or 2 days before the recording session, each bat was anesthetized with Nembutal (45–50 mg/kg b.w.), and the flat head of a 1.8-cm nail was glued onto the exposed skull with acrylic glue and dental cement. The exposed tissue was treated with an antibiotic (Neosporin) to prevent inflammation. The bat was administered a neuroleptanalgesic, Innovar-Vet (fentanyl 0.04 mg/kg b.w. droperidol 2 mg/kg b.w.) and placed inside a bat holder made of wire mesh that was suspended in an elastic sling inside a custom-made double-wall soundproof room with an ambient temperature of 28–30°C. The ceiling and inside walls of the room were covered with 8-cm convoluted polyurethane foam to reduce echoes.

After fixing the head of the anesthetized bat with a set screw, small holes (diameter, 200–500 μm) were drilled in the skull above the auditory midbrain, the inferior colliculus (IC), for orthogonal insertion of 2 M NaCl glass pipette electrodes (tip diameter <1 μm, impedance: 5–10 MΩ) to record sound activated responses. Additional doses of Innovar-Vet were administered during later phases of recording if the bats showed any signs of discomfort. A local anesthetic (lidocaine) was applied to the open wound area to reduce any possible pain. The recording depth was read from the scale of a microdrive (David Kopf Instrument, model 640, Tujunga, CA, United States). A common indifferent electrode (silver wire) was placed in the nearby temporal muscle. Each bat was used for one to five recording sessions on separate days and each recording session typically lasted for 2–6 h. An individual bat could be used several times after noise exposure because they can maintain hearing sensitivity after exposure to intense noise (Hom et al., 2016; our unpublished data from *H. pratti*). Thus, a minimal number of bats were used to obtain sufficient data for this study.

### Acoustic Stimulation

The background white noise was generated digitally using Tucker-Davis technologies (TDT, Alachua, FL, United States) system III hardware and OpenEX software. The noise was presented under acoustic free-field conditions through an electrostatic speaker (ED1, TDT). White noise was generated continuously and attenuated to a calibrated level of 70 dB SPL around the bat’s pinna with a 1/4-inch microphone (4939, B&K, Narum, Denmark).

For acoustic stimulation, continuous sine waves from a function generator (33500B, Agilent, Santa Clara, CA, United States) were formed into pure tone pulses or bursts (10 ms with rise-decay time of 0.5 ms, delivered at two pulses/s, hereafter identified as CF sound) by a custom-made tone burst generator driven by a stimulator (Master 8, AMPI, Jerusalem, Israel). The sounds were amplified after passing through a decade attenuator (LAT45, Leader, Kohokuku, Japan) before they were fed into a small loudspeaker (AKG model CK 50, 1.5 cm in diameter, 1.2 g, frequency response 1–100 kHz). The loudspeaker was placed 20 cm away from the animal’s ear and 30°contralateral to the recording site. Calibration of the loudspeaker was conducted using a 1/4-in. microphone (4939, B&K, Denmark) placed at the animal’s ear using a measuring amplifier (2610, B&K, Denmark). The output of the loudspeaker was expressed in decibel SPL in reference to the 20-μPa root mean square. A frequency-response curve of the loudspeaker was plotted to determine the maximal available sound amplitude at each frequency. The maximal stimulus amplitude ranged from 110 to 125 dB SPL between 10 and 80 kHz but dropped off to 80 dB SPL almost linearly from 80 to 100 kHz.

After isolating a neuron with 10-ms CF sounds, its response was amplified (ISO-DAM, WPI, Sarasota, FL, United States), band-pass filtered (Krohn-Hite 3500, Oceanside, CA, United States), and then passed through a window discriminator (WPI 121) before being sent to an oscilloscope (TDS210, Tek, Beaverton, OR, United States) and an audio monitor (Grass AM9, Warwick, RI, United States). The threshold of a neuron at its responsive frequency was audio-visually determined by changing the sound amplitude. At each responsive frequency, the threshold was defined as the amplitude which averagely elicited a 50% response probability to 16 stimuli. The sound frequency that elicited the neuron’s response at the lowest amplitude was defined as the BF. The threshold at the BF was defined as the MT. Whenever possible, the response properties for each IC neuron were studied under silence control and 70-dB SPL background white noise conditions which were presented at random. The neuron’s response also was sent to a computer (Kaitian 4600, Lenovo, China) to acquire the peristimulus-time (PST) histograms (bin width: 250 μs, sampling period: 145 ms) for 32 sound presentations. The PST histograms showed the neurons’ temporal discharge patterns to the sound stimuli. The total number of spikes in each histogram was used to quantify each neuron’s response under every stimulus condition.

The frequency tuning curves (FTCs) for the IC neurons were measured using combinations of sound frequency and amplitude that elicited a 50% response probability from the neuron (i.e., MT response). The frequency sensitivities of the FTCs was studied by measuring the *Q*_10 dB_ values, which were obtained by dividing the BF by the bandwidth (BW) of the FTC at 10 dB above the MT. Neurons with higher *Q*_10 dB_ values exhibited higher-frequency sensitivities than neurons with lower *Q*_10 dB_ values. The frequency selectivity of an FTC was expressed by the BW of FTC at 10 dB (BW10) and 30 dB (BW30) above MT. A neuron with a narrow BW exhibited higher-frequency selectivity than a neuron with a broad BW. The frequency selectivity was also studied by measuring the iso-level frequency spikes curve (FSC) with the number of spikes discharged to the stimulation at selected frequencies. Usually, selected frequencies were in 1 kHz step size decreasing or increasing from each neuron’s BF until the neuron had no response. However, if a neuron exhibited very sharp frequency tuning, 0.5 or 0.1 kHz step size was used. The amplitude of the stimulation was at 20 dB above each neuron’s MT. The frequency selectivity was expressed with a BW of each iso-level FSC at 50% (BW50%) and 75% (BW75%) of the maximum number of spikes. An iso-level FSC with a small BW had higher selectivity than an iso-level FSC with a large BW.

### Data Analysis

The data were processed and plotted using Sigmaplot, version 10.0 (Systat Software, San Jose, CA, United States). The results were expressed as means ± standard deviation (means ± SD) and then statistically analyzed using SPSS, version 13.0 (SPSS, Chicago, IL, United States). In all tests, *p* < 0.05 was considered statistically significant.

## Results

The pulses of *H. pratti* were recorded before, during, and after 70-dB SPL background white noise. A total of 1,036 high-quality pulses were recorded from four bats (bat1, 291; bat2, 225; bat3, 170; bat4, 350). Then, we carried out electrophysiology recordings. The MT and BF were obtained from 68 neurons under silent control and background noise conditions. However, only 55 neurons were studied with the frequency selectivity in both conditions due to loss of neurons during the study.

### Effects of Background White Noise on the Spectral Composition of *Hipposideros pratti* Echolocation Pulses

**FIGURE 1:**
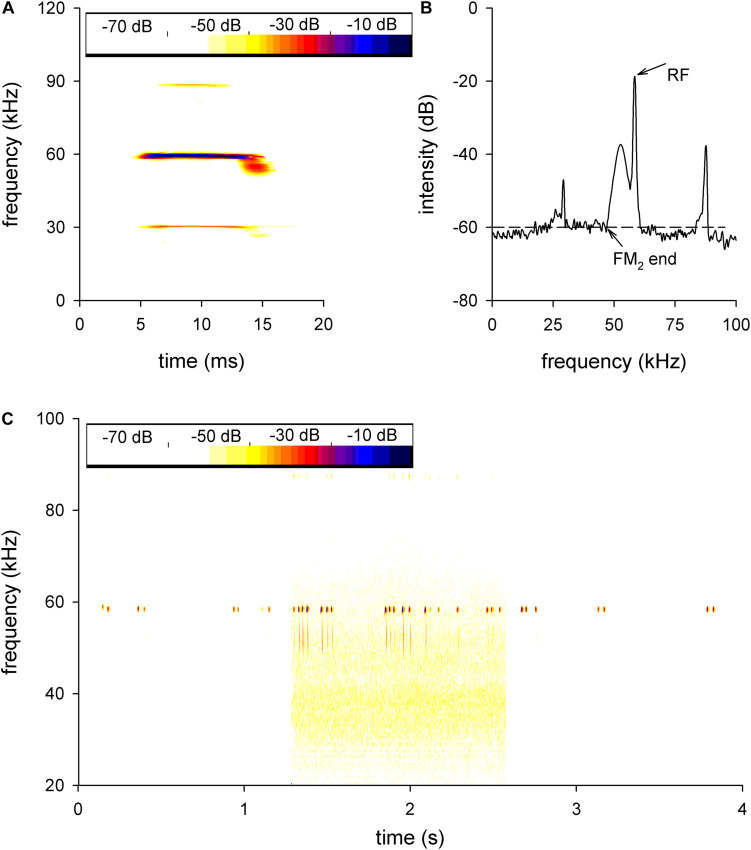
The echolocation call of *Hipposideros pratti*. Sonogram **(A)** and power **(B)** spectrum of the echolocation call of *H. pratti*. H1, first harmonic; H2, second harmonic; H3, third harmonic. The second harmonic is the predominant component of the echolocation call of *H. pratti*. The dashed line in panel **(B)** shows the method of analysis for the FM_2_ end frequency for *H. pratti*. RF, resting frequency; FM_2_, frequency modulation of the H2. **(C)** A representative echolocation trail of a bat before, during, and after exposure to background white noise, showing the nearly constant RFs and increased FM_2_ bandwidth during the background noise. The yellow background is due to the noise.

The echolocation pulses of *H. pratti* typically consisted of three harmonics (H1 to H3). Each harmonic included a CF component followed with a brief downward FM sweep (Figure 1A). The second harmonic (H2) presented the highest amplitude (Figures 1A,B) and was thought to be the predominant one. Therefore, we only analyzed the H2 in this study. The CF frequency of the H2 emitted while the bat was at rest was called the RF (Figure 1B). The FM_2_ was the FM component of H2, its start frequency was the RF, and the FM_2_ end frequency was set as the frequency with the first −60 dB amplitude drop (Figure 1B).

Figure 1C shows a representative echolocation trail before, during, and after applying the background white noise conditions. The bat called at a relatively low pulse rate in the experimental anechoic cage before the noise condition (Figure 1C, left). During the background white noise condition, the bat increased the pulse rates and FM_2_ ranges while keeping the RF nearly unchanged (Figure 1C, middle). After the noise, the echolocation pulse was similar to the pulse given before the noise condition (Figure 1C, right).

**FIGURE 2:**
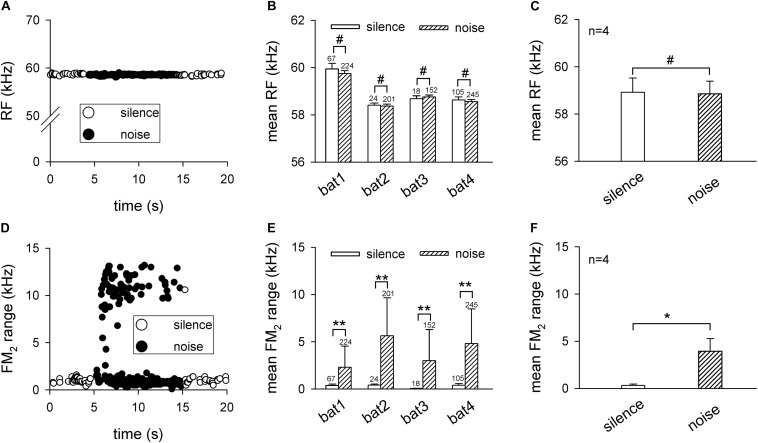
The noise influence on the second harmonic of the echolocation call of *Hipposideros pratti* in the frequency domain. **(A)** The RF of a representative echolocation call trial from a bat before (0–5 s), during (5–15 s), and after (15–20 s) exposure to background white noise, showing a nearly constant RFs during the background noise. **(B)** Comparison of the each bat’s RF in silence and noise conditions. Although the RF of each bat varied a small amount, all bats kept the RF unchanged in the noise condition. **(C)** Comparison of the mean RF for all bats between silence and noise conditions, showing that *H. pratti* kept the RF unchanged in silence and noise conditions. **(D)** The FM_2_ range of a representative echolocation call trial from a bat before (0–5 s), during (5–15 s), and after (15–20 s) background white noise, showing that the FM_2_ range of some calls increased during the background noise. **(E)** Comparison of each bat’s FM_2_ range in silence and noise conditions, showing that all bats significantly increased the FM_2_ range in the noise condition. **(F)** Comparison of the mean FM_2_ range for all bats between silence and noise conditions, showing that *H. pratti* significantly increased the FM_2_ range in noise conditions. RF, resting frequency. FM_2_, frequency modulation of the H2. ^#^*p* > 0.05; *p < 0.05; **p < 0.01.

The effects of background white noise on the RF and FM_2_ range were quantified in Figure 2. The RF was constant between silence and the background white noise condition in the representative trial (Figure 2A). Statistical analysis revealed that the noise did not shift the RF for all four bats (bat1: 59.95 ± 0.23 vs. 59.75 ± 0.12 kHz, *p* > 0.05; bat2: 58.41 ± 0.09 vs. 58.37 ± 0.08 kHz, *p* > 0.05; bat3: 58.68 ± 0.13 vs. 58.76 ± 0.08 kHz, *p* > 0.05; and bat4: 58.63 ± 0.13 vs. 58.56 ± 0.09 kHz, *p* > 0.05, all analyzed using the Friedman’s ANOVA test; Figure 2B). In addition, the RF of all four bats also did not differ significantly between the silence and noise condition (58.92 ± 0.60 vs. 58.86 ± 0.53 kHz, *p* > 0.05, paired *t* test; Figure 2C). Differing from the RF, the bats greatly increased the FM_2_ range of a few pulses while keeping the others nearly unchanged (Figure 2D). Statistical analysis demonstrated that the FM_2_ range significantly increased during the background white noise condition for the four bats (bat1: 0.38 ± 0.15 vs. 2.31 ± 2.23 kHz, *p* < 0.01; bat2: 0.41 ± 0.12 vs. 5.64 ± 4.02 kHz, *p* < 0.01; bat3: 0.06 ± 0.02 vs. 3.00 ± 3.30 kHz, *p* < 0.01; and bat4: 0.41 ± 0.17 vs. 4.81 ± 3.67 kHz, *p* < 0.01, all analyzed using the Friedman’s ANOVA test; Figure 2E). The averaged data for the four bats indicated that the FM_2_ range during the noise condition was significantly wider than that during silence condition (0.32 ± 0.15 vs. 3.94 ± 1.34 kHz, *p* < 0.05, paired *t* test; Figure 2F). Note that our data demonstrated the increase in the FM_2_ range during the noise condition. The exact FM_2_ range might be underestimated because we used the low-gain control to record the echolocation pulses to avoid saturation of the recording system. Taken together, the 70-dB SPL background white noise did not shift the RF while it significantly increased the FM_2_ range of the bat’s echolocation pulses.

### Effects of Background White Noise on the BFs of Midbrain Neurons in *Hipposideros pratti*

**FIGURE 3:**
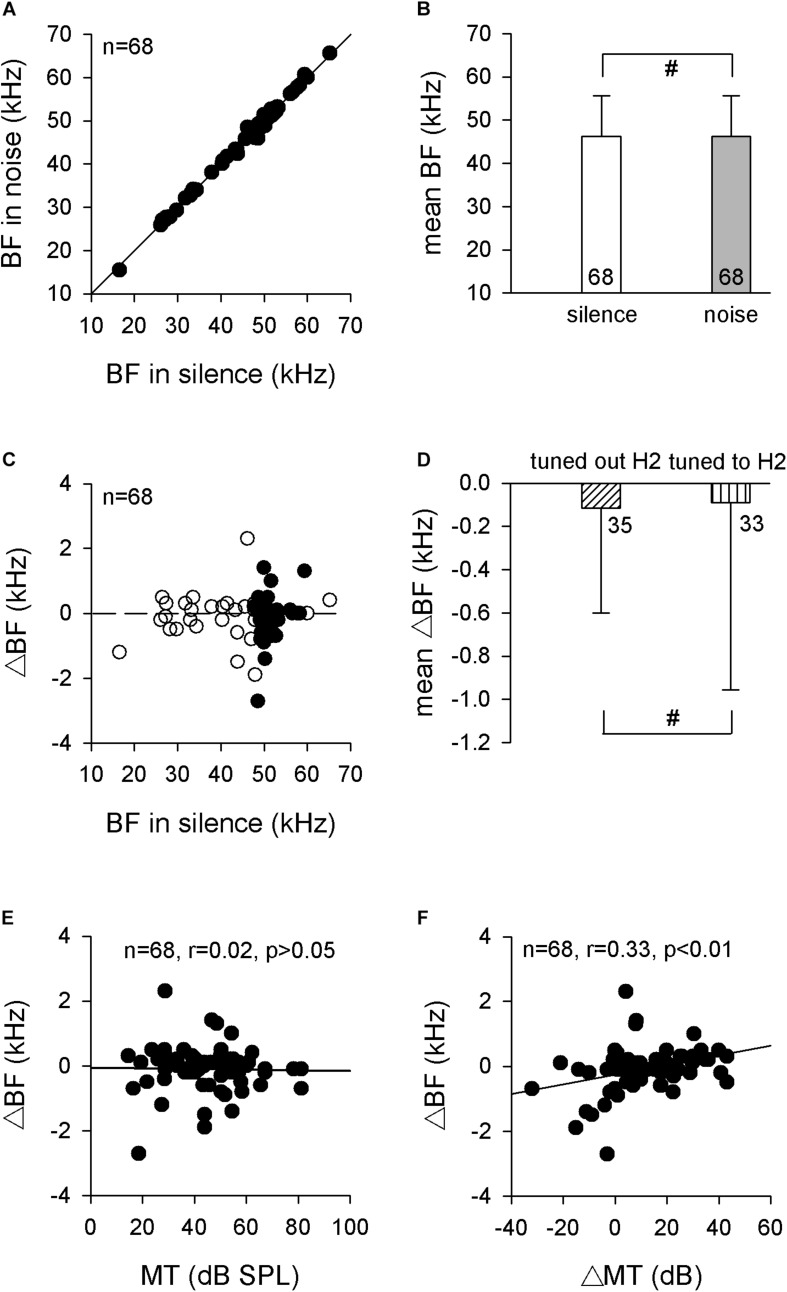
The BFs of auditory midbrain neurons in silence and noise conditions. **(A)** The distribution of BF in noise compared with BF in silence. The solid line is the line of equality. **(B)** Comparison of the mean BF in silence and noise conditions. **(C)** The distribution of ΔBF against BF in the silence condition. The horizontal dashed line is the zero line. Filled circle, neurons tuned to H2; unfilled circle, neurons tuned out H2. **(D)** Comparison of the mean ΔBFs between neurons that tuned out H2 and neurons that tuned to H2. **(E)** The distribution of ΔBF against MT. Linear-regression analysis showed no significant correlation. **(F)** The distribution of ΔBF against ΔMT. Linear-regression analysis showed a significant positive correlation. BF, best frequency. H2, the second harmonic. MT, minimal threshold. ^#^*p* > 0.05.

Since the RF was constant between silence and the 70-dB SPL white noise conditions, it was reasonable to speculate that the auditory midbrain neurons should hold their frequency analysis to process the pulse frequency during the noise. We tested this supposition by studying the BFs of auditory midbrain neurons in both silence and noise conditions firstly. Sixty-eight single neurons were isolated, and their BFs in silence increased linearly with increasing recording depth (*r* = 0.515, *p* < 0.001, Pearson’s correlation), which suggested that these neurons were topologically located in the central nucleus of the IC (ICC). The BF distribution during noise compared with BFs in silence revealed that most neurons had similar or the same BFs in both conditions, as shown by the dots around the line of equality in Figure 3A. Statistical analysis revealed that the 70-dB SPL background white noise had no significant influence on the BFs (46.3 ± 9.3 vs. 46.2 ± 9.4 kHz, *p* > 0.05, paired *t* test; Figure 3B).

Although the mean BFs remained unchanged during noise conditions, we also observed that the BFs of some neurons exhibited slight differences between silence and noise conditions, as seen by some dots diverged from the line of equality (Figure 3A). Our previous study indicated that the 70-dB SPL background white noise increased the MTs of neurons in the ICC, and the ΔMTs were comparable with the pulse intensity increments induced by the same noise (Cui et al., 2021). Therefore, we calculated the changes in BFs (ΔBFs), which were defined as the BFs during the noise condition minus the BFs during silence. However, the ΔBFs were almost evenly distributed around the zero line, although H2 neurons exhibited greater variation (Figure 3C). The frequency spectrum for H2 was determined by the echolocation pulses recorded before each electrophysiological recording. Therefore, the mean of the ΔBFs for neurons that tuned to the H2 were not significantly different from the mean ΔBFs for neurons that tuned out the H2 (−0.09 ± 0.86 vs. −0.11 ± 0.49 kHz, *p* > 0.05, unpaired *t* test; Figure 3D). Because the ICC is known to exhibit fibro-dendritic laminae (Oliver and Morest, 1984), the present results suggested that the mean BFs in each lamina remained constant between the silence and background white noise conditions.

We also studied the possible correlation between the ΔBFs and MTs of these neurons using linear regression analysis of the scatter plots of the ΔBFs against the MTs. The MTs of IC neurons with a similar ΔBF varied over a wide range, and the linear regression analyses of these plots did not reveal significant correlations between the MT and ΔBF (*r* = 0.02, *p* = 0.849, Pearson’s correlation; Figure 3E). However, the ΔBFs significantly increased with ΔMTs such that the high ΔMT neurons tended to have higher ΔBFs than low ΔMT neurons (*r* = 0.33, *p* < 0.01, Pearson’s correlation; Figure 3F).

### Effect of Background White Noise on the Frequency Sensitivity of Midbrain Neurons in *Hipposideros pratti*

**FIGURE 4:**
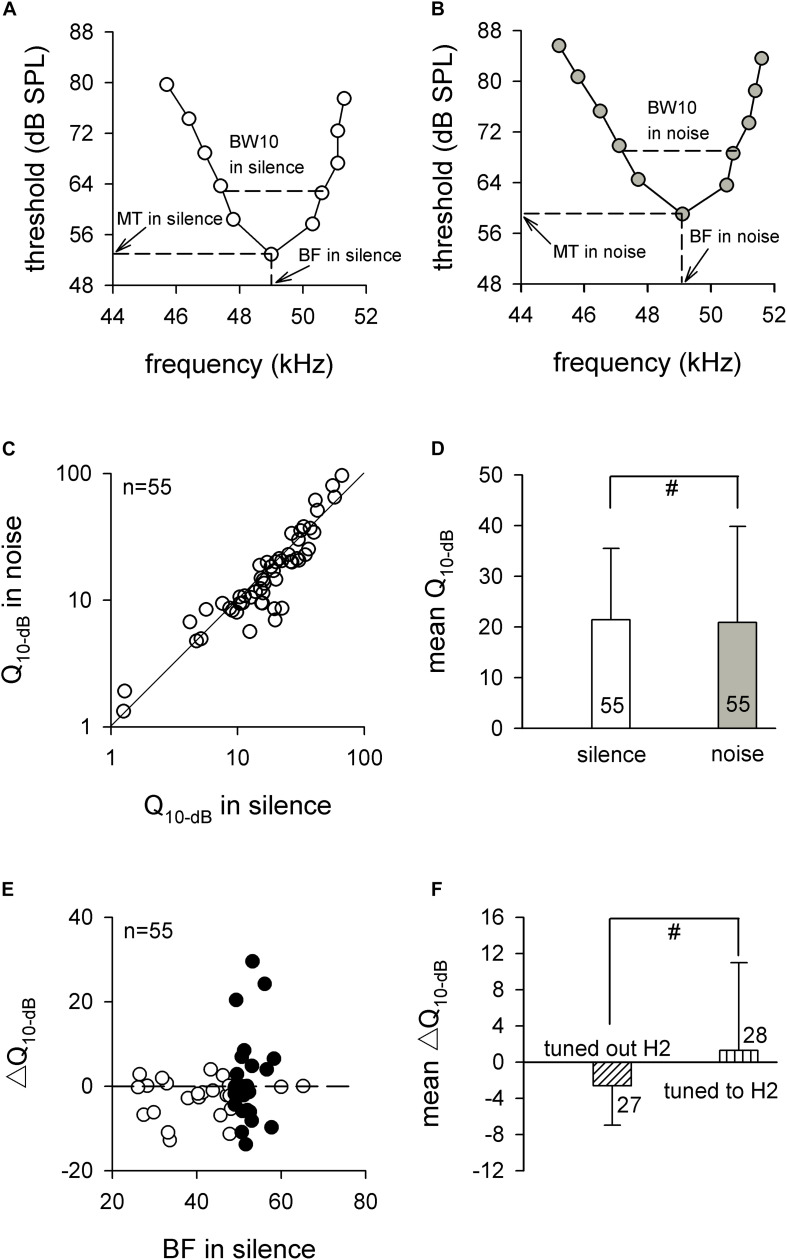
The frequency sensitivities of auditory midbrain neurons in silence and noise conditions. **(A,B)** A representative frequency tuning curve for auditory midbrain neurons in silence **(A)** and noise **(B)** conditions. The dashed line shows the method for obtaining BF, MT, and BW10. **(C)** The distribution of *Q*_10 dB_ in noise condition compared with *Q*_10 dB_ in silence. The solid line is the line of equality. **(D)** Comparison of the mean *Q*_10 dB_ between silence and noise conditions. **(E)** The distribution of Δ*Q*_10 dB_ against BF in silence. The horizontal dashed line is the zero line. Filled circle, neurons tuned to H2; unfilled circle, neurons tuned out H2. **(F)** Comparison of the mean Δ*Q*_10 dB_ between neurons that tuned out H2 and neurons that tuned to H2. BF, best frequency. MT, minimal threshold. BW, bandwidth. ^#^*p* > 0.05.

To further assess the frequency analysis of auditory midbrain IC neurons, we studied their frequency sensitivity by measuring the *Q*_10 dB_ values of the FTCs, which were obtained by dividing the BF by the BW of the FTC at 10 dB above the MT in silence and background white noise conditions (Figures 4A,B). The effect of the background white noise on the frequency sensitivities of the ICC neurons was studied by comparing the *Q*_10 dB_ in noise and silence. As shown in Figure 4C, the *Q*_10 dB_ values in noise for most neurons were similar to the *Q*_10 dB_ values in silence, most of the data points were distributed at or near the diagonal line (line of equality). The statistical analysis showed that the *Q*_10 dB_ values in noise were not significantly different from the *Q*_10 dB_ values in silence (20.9 ± 19.0 vs. 21.4 ± 14.1, *p* > 0.05, paired *t* test; Figure 4D).

We calculated the Δ*Q*_10 dB_ by subtracting the *Q*_10 dB_ in noise from the *Q*_10 dB_ in silence. The distribution of Δ*Q*_10 dB_ against the BF in silence is seen in Figure 4E. Although the neurons tuned to the H2 exhibited greater variation, the data points were nearly even distributed around the horizontal dashed line (Figure 4E), and there was no significant difference between the averaged Δ*Q*_10 dB_ for neurons that tuned out H2 and averaged Δ*Q*_10 dB_ for neurons that tuned to H2 (−2.6 ± 4.4 vs. 1.3 ± 9.7, *p* > 0.05, unpaired *t* test; Figure 4F). Taken together, these results demonstrated that the background white noise had no significant influence on the mean Δ*Q*_10 dB_ in each of the fibro-dendritic laminae, indicating that the effect of the noise on the frequency sensitivities of ICC neurons might not depend on the fibro-dendritic laminae.

### Effect of Background White Noise on the Frequency Selectivity of Midbrain Neurons in *Hipposideros pratti*

**FIGURE 5:**
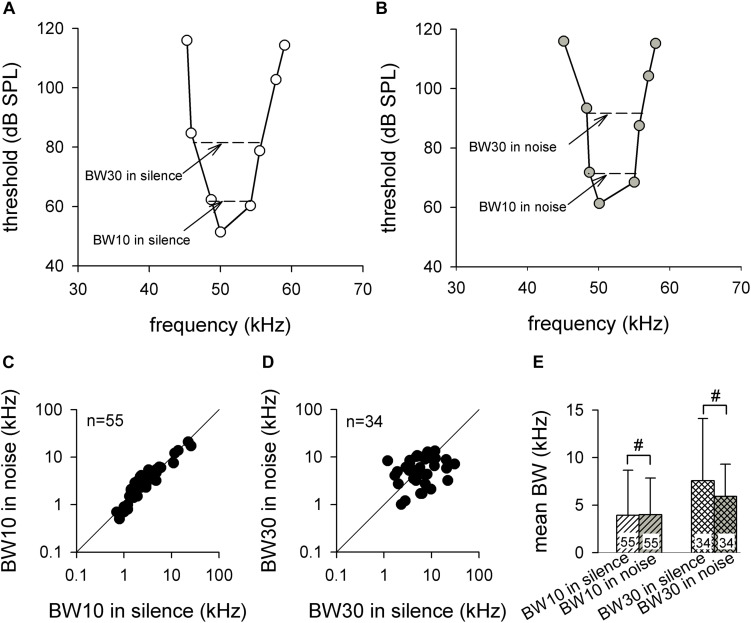
The frequency selectivities of auditory midbrain neurons in silence and noise conditions. **(A,B)** A representative frequency tuning curve for auditory midbrain neurons in silence **(A)** and noise **(B)** conditions. The dashed line shows the method for obtaining BW10 and BW30. **(C,D)** The distribution of BW10 **(C)** and BW30 **(D)** in noise compared with silence. The solid line is the line of equality. **(E)** Comparison of the mean BW10 (left) and BW30 (right) between silence and noise conditions. BW, bandwidth. ^#^*p* >0.05.

The neuronal frequency analysis is occasionally studied using frequency selectivity, which was expressed by the BW of FTC at 10 dB (BW10) and 30 dB (BW30) above MT (Christensen et al., 2019; Lee et al., 2019). Therefore, we studied the frequency selectivity by measuring the BW10 and BW30 in silence and background white noise conditions (Figures 5A,B). Because the MTs of some neurons were too high to give a MT + 30 stimulation or loss of neurons during the FTC recording, we only obtained BW30 from 34 ICC neurons in both silence and noise conditions. Figure 5C shows the scatter plot for BW10 in noise against BW10 in silence. Most of the data points were evenly distributed around the line of equality. The statistical analysis demonstrated that the noise exhibited no significant influence on BW10 (3.9 ± 4.7 vs. 4.0 ± 3.8, *p* > 0.05, paired *t* test; Figure 5E, left). Similarly, most of the data points of the scatter plot revealed that the BW30 in noise against BW30 in silence also were primarily distributed evenly around the line of equality, although with greater variation (Figure 5D). The statistical analysis revealed no significant difference between the BW30 in silence and the BW30 in noise (7.6 ± 6.5 vs. 5.9 ± 3.4, *p* > 0.05, paired *t* test; Figure 5E, right). We further studied the frequency selectivity by measuring the BW50% and BW75% of the iso-level FSC (see section “Materials and Methods” for details) in 15 auditory ICC neurons. The results showed that both BW50% (4.1 ± 1.9 vs. 4.7 ± 2.6, *p* > 0.05, paired *t* test) and BW75% (2.0 ± 0.9 vs. 2.4 ± 1.2, *p* > 0.05, paired *t* test) remained constant between the silence and noise conditions.

**FIGURE 6:**
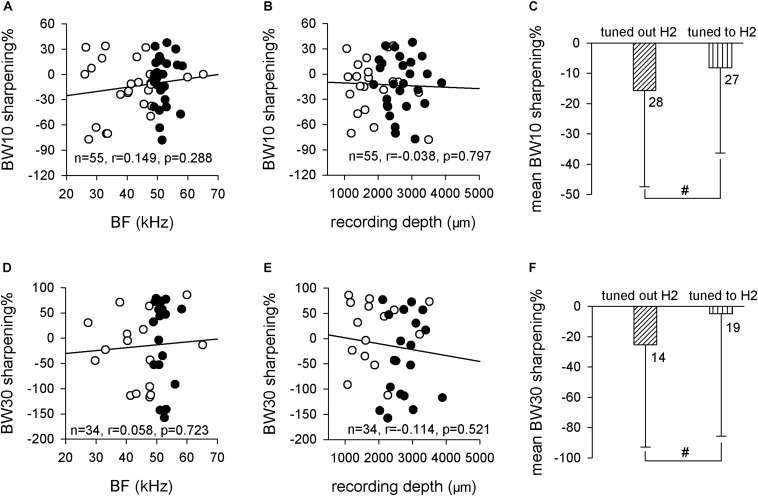
The relationship between BW sharpening% and neuron’s BF or recording depth. **(A,B)** The distribution of BW10 sharpening% against BF **(A)** and recording depth **(B)**. Linear-regression analysis did not indicate any significant correlation. **(C)** Comparison of the mean BW10 sharpening% between neurons that tuned out H2 and neurons that tuned to H2. **(D,E)** The distribution of BW30 sharpening% against BF **(D)** and recording depth **(E)**. Linear-regression analysis did not reveal any significant correlation. **(F)** Comparison of the mean BW30 sharpening% between neurons that tuned out H2 and neurons that tuned to H2. Filled circle, neurons tuned to H2; unfilled circle, neurons tuned out H2. ^#^*p* > 0.05.

The sharpening% was used to analyze the frequency selectivity change quantitatively, which was defined as the value of the BW in noise minus the BW in silence divided by the BW in silence. Linear regression analysis showed the both BW10 sharpening% and BW30 sharpening% exhibited no linear relationship with both BF (BW10 sharpening%: *n* = 55, *r* = 0.149, *p* = 0.288, Pearson’s correlation; Figure 6A. BW30 sharpening%: *n* = 34, *r* = 0.058, *p* = 0.723, Pearson’s correlation; Figure 6D) and recording depth (BW10 sharpening%: *n* = 55, *r* = −0.038, *p* = 0.797, Pearson’s correlation; Figure 6B. BW30 sharpening%: *n* = 34, *r* = −0.114, *p* = 0.521, Pearson’s correlation; Figure 6E). The sharpening% was not significantly different between neurons that tuned out H2 and neurons that tuned to H2 for both BW10 (−15.7 ± 31.8 vs. −8.2 ± 28.1, *p* > 0.05, unpaired *t* test; Figure 6C) and BW30 (−25.3 ± 67.5 vs. −4.8 ± 80.8, *p* > 0.05, unpaired *t* test; Figure 6F). Therefore, these results suggested that the effect of background white noise on the frequency selectivities of ICC neurons might not depend on the fibro-dendritic laminae.

## Discussion

The present data revealed that 70-dB SPL background white noise did not shift the RFs of echolocation pulses of *H. pratti* but did increase the FM_2_ range. Single-cell extracellular recording demonstrated that the background noise had no significant effect on the BFs, frequency sensitivities, and frequency selectivities of the auditory midbrain ICC neurons. Our recently published study with the same bat species indicated that the background noise could increase the pulse intensity, raise the MT of the ICC neurons, and sharpen their sound amplitude tuning (Cui et al., 2021). These results indicated that the neuronal response properties could be modified to adapt to processing the vocal in background noise condition, and the bats were able to adjust pulse amplitude and frequency independently.

### Constant RFs in the Noise Condition

Many vertebrates exhibit the Lombard effect in noisy environments, including echolocating bats (Luo et al., 2018). Our recently published results showed that the pulse intensity of *H. pratti* increased robustly with background white noise conditions (Cui et al., 2021). However, the bats kept the RFs unchanged with 70-dB SPL background white noise (Figures 1,2), which was in agreement with a recent study with 40–64 dB SPL background white noise (Lu et al., 2020). Thus, the short CF-FM bats might keep their RF unchanged in noise conditions, which was different from the long CF-FM bats (Hage et al., 2013). Since the RF was thought to play an essential role during echolocation, the auditory system was specialized to accurately process the RF [reviewed by Ulanovsky and Moss (2008) and Schnitzler and Denzinger (2011)]. Therefore, the constant RF required constant neuronal frequency analysis in conditions with noise. Our electrophysiological data showed that the ICC neurons exhibited constant BFs, frequency sensitivities, and frequency selectivities (Figures 3–6). Differing from the constant RF in noise conditions, the FM_2_ range increased significantly (Figures 1, 2). Because the echolocating bats increased the FM range in the terminal phase of their echolocation, the increasing FM_2_ range was thought to be suited for target distance processing (Schnitzler and Kalko, 2001). However, the increasing FM_2_ range might also be an inevitable by-product of the increasing pulse intensity.

The constant neuronal frequency analysis might not be restricted to H2 neurons. We proposed that the constant frequency analysis properties across all fibro-dendritic laminae kept the RF analysis constant. The ICC is defined by the presence of fibro-dendritic laminae, and there are lots of local circuitry linking neurons in the same lamina and across laminae [reviewed by Ito and Malmierca (2018)]. Therefore, changes in frequency analysis properties in one lamina might alter the local circuitry of the RF neurons and consequently change the RF analysis. Thus, the neuronal frequency analysis in noise conditions was tightly linked to the RF of bat’s echolocation pulses in the same conditions. In the present study, we showed that the bats could hold their midbrain neuronal frequency analysis in noise conditions, but the frequency analysis can be shifted by attention, context, and so on (O’Connell et al., 2014).

### Strategies for Overcoming Noise Interference in Echolocating Bats

During echolocation, the bats must continuously emit pulses to assess the changing environment in real time. Bats typically live and forage in groups, and their hunting environments, especially for CF-FM bats, are quite complex (Corcoran and Moss, 2017). Besides the noise from the bat’s everyday activities (biotic noise), there is also abiotic noise; for example, noise caused by wind, rain, and flowing water (Brumm et al., 2004). Taken together, the living habitat of the echolocating bat is very noisy. Bats employ a number of strategies to overcome the influence of noise. Some of the strategies are common to vertebrates or mammals, and other strategies are species specific. Echolocating bats share some strategies used by other vocal animals to overcome noise interference. These strategies include evolutionary responses and short-term adaptations (Brumm et al., 2004). Previous studies have demonstrated that bats exhibit the Lombard effect in noise conditions (Simmons et al., 1978; Tressler and Smotherman, 2009; Hage et al., 2013; Luo et al., 2017a; Cui et al., 2021), even in 2-week-old bats (Luo et al., 2017b). However, the pulse frequency adjustment in noise was not as widespread as amplitude, which will be discussed later.

Echolocating bats also exhibit several specific strategies in response to noise interference. Usually, the echolocation pulses are ultrasound signals, which are different from most other animals’ calls and abiotic sounds. Thus, echolocating bats avoid interference from most biotic and abiotic sounds. Moreover, the echolocating bats utilize several specialized neurons in their auditory system, including the delay-tuned combination-sensitive neurons (also called FM/FM neurons) and CF/CF combination-sensitive neurons (Suzuki and Suga, 2017; Suga, 2018), which are thought to play critical roles in obtaining relative distance and velocity information, respectively, from their targets. Bats could use these combination-sensitive neurons to “tag” their own calls and process only its own echoes, ignoring the pulses and echoes from other bats (Ulanovsky and Moss, 2008). Thus, the echolocating bats use both common and species-specific strategies to overcome noise interference.

### Variation of Noise-Induced Pulse Changes in the Frequency Domain

Many animals can adjust their call to maintain the signal-to-noise ratio in noise conditions. Previous studies have shown that most animals increase their call intensity, although a few species do not exhibit intensity changes (Brumm and Zollinger, 2017; Zhao et al., 2018). However, the observed adjustments in the frequency domain are quite variable, depending on both the frequency spectrum of the noise and the species studied. For example, low-frequency background noise usually increases the minimum frequency of the acoustic signals in birds (Reichard et al., 2020). Water flow noise also increases the call frequency in frogs (Shen and Xu, 2016), even without the Lombard effect (Zhao et al., 2018). Studies on whale’s show that they tend to shift the peak energy frequency higher in noise conditions regardless of the noise frequency spectrum (Au et al., 1985; Lesage et al., 1999; Rendell and Gordon, 1999). In human speech, a rise in fundamental frequency is accompanied by the Lombard effect (Lu and Cooke, 2008).

The precise pulse frequency control and analysis by echolocating bats have been extensively studied. In response to noise interference from conspecifics, bats actively modify the spectral characteristics of their pulses, a well-known behavior called the jamming avoidance response (JAR) (Bates et al., 2008). However, some studies indicated no JAR occurred with severe acoustic interference (Amichai et al., 2015). There are only a few studies on echolocating bats using background broad-band noise. Tressler and Smotherman (2009) found that *Tadarida brasiliensis mexicana* significantly increased the start frequency of the pulse and decreased the end frequency of the pulse in noise spanning a range of 15–100 kHz. However, Luo and Wiegrebe (2016) showed that *Phyllostomus discolor* consistently decreased the spectral parameters of their pulses in noise, whose spectrum ranged from 10 to 90 kHz. Among long CF-FM bats, only the *R. ferrumequinum* was studied using band pass-filtered noise (BW 20 kHz), and the influence depended on the centered frequency of the noise. The bats kept the RF unchanged when the noise was centered at 500 Hz higher than the RF and increased the RF when the noise centered at or below the RF (Hage and Metzner, 2013; Hage et al., 2013). For the short CF-FM bat, this study and a recent study (Lu et al., 2020) showed that *H. pratti* and *Hipposideros armiger* exhibited a relatively stable RF in white noise. Because these studies worked on different family of bat species and broadcasted noise of distinct frequency ranges, the driving factors for the reported differences in noise-induced modifications of the RF were unclear. Additional comparative studies across different species using the same type of noise are needed to answer this question.

Studies on how animals adjust their calls in noise conditions help reveal the neurophysiological basis of audiovocal integration (Luo et al., 2018). The results stated above suggest that, at least for some animals, call frequency and intensity are controlled independently. Additional study of the neural basis for independent control of call frequency and intensity may provide a reference for understanding the neural underpinnings of audiovocal integration.

## Data Availability Statement

The original contributions presented in the study are included in the article/supplementary material, further inquiries can be directed to the corresponding author/s.

## Ethics Statement

The animal study was reviewed and approved by Institutional Animal Care and Use Committee of Central China Normal University.

## Author Contributions

ZF contributed to the conception and design of the study, acquisition, analysis, interpretation of the data, and drafting the manuscript. GZ and ZC contributed to acquisition, analysis, and interpretation of the data and revising the draft critically for important intellectual content. JW, BJ, DZ, LL, JT, and QC contributed to revising the draft critically for important intellectual content. All authors have approved the final version of the manuscript and agreed to be accountable for all aspects of the study. The study was conducted in the laboratory at Central China Normal University. All persons designated as authors qualify for authorship and all those who qualify for authorship are listed.

## Conflict of Interest

The authors declare that the research was conducted in the absence of any commercial or financial relationships that could be construed as a potential conflict of interest.
